# Clinical comparison of four commercial MRI phantoms on a 0.35T MR‐linac using a standard clinical TRUFI sequence at a single institution

**DOI:** 10.1002/acm2.70247

**Published:** 2025-09-09

**Authors:** Mateb Al Khalifa, Tianjun Ma, Haya Aljuaid, Siyong Kim, William Y. Song

**Affiliations:** ^1^ Department of Radiation Oncology Virginia Commonwealth University Richmond Virginia USA; ^2^ Department of Radiation Medicine Northwell Health Lake Success New York USA

**Keywords:** acceptance, commissioning, large FOV phantom, MRgRT, quality assurance

## Abstract

**Purpose:**

Real‑time magnetic resonance–guided radiation therapy (MRgRT) integrates MRI with a linear accelerator (Linac) for gating and adaptive radiotherapy, which requires robust image‑quality assurance over a large field of view (FOV). Specialized phantoms capable of accommodating this extensive FOV are therefore essential. This study compares the performance of four commercial MRI phantoms on a 0.35 T MR‑Linac.

**Methods:**

Four phantoms: the IBA QUASAR Insight Phantom, the IBA QUASAR MRID^3D^ Geometric Distortion Phantom, the MagPhan Phantom, and the Sun Nuclear Large Field MRI Distortion Phantom, were evaluated on a 0.35 T MR‐Linac using the TRUFI clinical sequence. Mean distortions were measured at gantry angles from 0° to 360° in 30° increments. Clinical imaging parameters (signal‐to‐noise ratio, uniformity, resolution via modulation transfer function, laser alignment, and twist) were also assessed where applicable.

**Results:**

The QUASAR MRID^3D^ Phantom enabled separate quantification of B_0_ and gradient‐induced distortions but showed a notable vertical offset, which did not affect comparisons with other phantoms. Both the MagPhan and QUASAR Insight phantoms provided mean distortion measurements as well as additional imaging parameters (e.g., signal‐to‐noise ratio, uniformity, resolution). The Sun Nuclear Phantom exhibited higher overall distortion values.

**Conclusion:**

All four phantoms successfully measured distortion at multiple gantry angles on a 0.35 T MR‐Linac. The QUASAR MRID^3D^ Phantom uniquely differentiated B_0_ from gradient distortions but required an offset adjustment. The MagPhan and QUASAR Insight phantoms offered comprehensive imaging metrics, while the Sun Nuclear Phantom exhibited relatively higher distortions.

## 1 INTRODUCTION

Magnetic resonance imaging (MRI) is widely used in radiotherapy for treatment planning because it provides superior soft‐tissue contrast compared to x‐ray imaging, enabling more precise delineation of tumor targets and organs at risk. Integrating an MRI system with a Linac enhances image‐guided radiotherapy (IGRT) by allowing real‐time observation of tumor changes and optimal normal‐tissue preservation, thereby supporting adaptive radiotherapy.^1^, ^2^, ^3^, ^4^, ^5^ An additional benefit is that MRI does not introduce extra ionizing radiation, unlike x‐ray gating, making it particularly appealing for IGRT.

Currently, three vendors provide clinically available MRI‐Linac systems at different field strengths: 0.35T MR‐Linac (ViewRay), 0.5T MR‐Linac Aurora RT (MagnetTx Oncology Solutions), and 1.5T MR‐Linac (Elekta Unity, Elekta Instrument AB).^6^, ^7^, ^8^ Both the 0.35T and 1.5T MR‐Linac designs use fixed cylindrical MRI magnets with the magnetic field perpendicular to the x‐ray beam, whereas the 0.5T MR‐Linac aligns the gantry and magnetic field parallel to the x‐ray beam.^9^ In the 0.35T system, B_0_ fluctuations have been observed, leading to imaging artifacts and isocenter shifts during gantry rotation, which affect image quality for gating and adaptive radiotherapy.^10^, ^11^ The 0.35T MR‐Linac contains a shielded Linac component integrated within the MRI, maintaining a 28 cm gap between the magnet assemblies. Its Linac and MRI isocenters coincide at a 90 cm source‐to‐axis distance (SAD). However, for the 1.5 T MR‐Linac, B_0_ homogeneity and geometric distortion remain constant with gantry angle,^9^ as the Linac parts are placed behind the MRI system, resulting in an SAD of 143.5 cm.

Geometric distortions arise from two main sources: the patient and the MRI‐Linac system.^12^, ^13^ Patient‐induced distortions stem from magnetic susceptibility and chemical shift effects, which are minimal and difficult to correct.^14^ System‐related distortions are predominantly linked to MRI hardware, including main magnetic field inhomogeneity, gradient uniformity, and linearity.^11^, ^15^, ^16^, ^17^, ^18^, ^19^, ^20^ Such distortions degrade image quality and may reduce the accuracy of gating and adaptive radiotherapy.

The severity of distortions depends on factors such as magnetic field strength, imaging sequence, and receiver bandwidth.^21^, ^22^, ^23^ At 0.35 T, the clinical mode sequence is a balanced steady‐state free precession (bSSFP), also known as TrueFISP (Siemens), Balanced FFE (Philips), or FIESTA (GE), termed TRUFI in this system. bSSFP offers high signal‐to‐noise ratio (SNR) and excellent temporal resolution,^24^, ^25^ but is highly sensitive to B_0_ fluctuations. These fluctuations can produce signal loss or dephasing (null bands), which are influenced by gantry rotation in the 0.35 T MR‐Linac.^11^ Moreover, an offset in the center frequency may shift the MRI isocenter, causing misalignment between the MRI and Linac isocenters.^10^ In the 0.35 T MR‐Linac, bSSFP is used for both real‐time imaging and general imaging (e.g., treatment planning, localization), with various clinical protocols that adjust field of view, slice thickness, and other parameters to optimize SNR.

Multiple commercial and in‐house phantoms exist to measure B_0_ fluctuations and other image‐quality metrics.^26^, ^27^, ^28^, ^29^ Nejad‐Davarani et al. introduced a large FOV phantom to assess gradient nonlinearity and overall distortions by examining frequency‐encoding directions in the anterior–posterior, left–right, and superior–inferior planes at multiple radial distances from the magnet's center.^30^ Kielbasa et al. assessed image uniformity and spatial integrity under conditions such as multileaf collimator (MLC) motion, static gantry position, and gantry rotation, using both a 24 cm NEMA phantom (Siemens) for uniformity and a linearity phantom (Fluke 76–907, HP Manufacturing) for spatial integrity.^31^ Lewis et al. employed the QUASAR Insight Phantom, imaging it in axial and sagittal planes, to evaluate geometric accuracy, spatial resolution, slice thickness, slice position, and image intensity uniformity.^32^ Hasler et al. studied geometrical distortions in clinical MRI sequences across multiple centers using the MagPhan phantom,^33^ while Marasini et al. investigated geometric distortions and isocenter shifts via the QUASAR MRID^3D^ phantom and Flock phantom at various gantry angles.^34^

In this study, we compare four commercial phantoms: QUASAR Insight Phantom, QUASAR MRID^3D^ Geometric Distortion Phantom, MagPhan Phantom, and Sun Nuclear Large Field MRI Distortion Phantom, in a single‐institution 0.35 T MR‐Linac. We examine each phantom's characteristics, measurement range, accuracy, and capabilities using the TRUFI clinical sequence mode.

## 2 MATERIALS AND METHODS

### 2.1 MRI phantoms

#### 2.1.1 IBA QUASAR MRgRT insight phantom

The QUASAR MRgRT Insight Phantom (Modus QA) is constructed from acrylic (PMMA) and filled with a T1 contrast mineral oil. It consists of two plates: one horizontal, intended for coronal imaging, and one upright, used for axial and sagittal scans. The horizontal (base) plate is rectangular, measuring 710 mm × 380 mm × 446 mm, while the upright plate is semi‐circular, with a diameter of 426 mm and a thickness of 58 mm. A coil bridge accessory is included to support and position the torso coil array securely.

This phantom can be used for both daily and periodic MRI quality assurance, providing parameters such as laser isocenter precision, distortion, ghosting, spatial resolution, slice thickness, and phased array coil uniformity and SNR. It can also facilitate beam checks using film cassettes or an ion chamber for daily or end‐to‐end tests, although these procedures lie beyond the scope of this study.

#### 2.1.2 IBA QUASAR MRID^3D^ geometric distortion phantom

The QUASAR MRID^3D^ Cylindrical Phantom is specifically designed to measure geometric distortions in MRI scans. Its physical dimensions are 394 mm in diameter and 391 mm in length, while the imaging dimensions are 368 mm in diameter and 321 mm in length. Shaped like a hollow cylinder with an internal volume of approximately 25 L, the phantom weighs 21 kg. It is constructed from acrylic and filled with a high T1 contrast mineral oil.

The phantom contains 1502 fiducials, uniformly spaced 18 mm apart. Of these fiducials, each 6 mm long, 1496 have a 3 mm diameter, and six have a 5 mm diameter. An insert at the center is shaped like a cuboid with dimensions of 30, 40, and 50 mm along the *X*, *Y*, and *Z* axes, respectively. This inserts aids localization and serves as a visual reference for the phantom's dimensions in the MRI reference frame.

To measure geometric distortion arising from both B_0_ and gradient nonlinearity, two scans are required, as recommended by the manufacturer. In the second scan, phase encoding was reversed (A→P switched to P→A) to facilitate separate B_0_ and gradient nonlinearity induced distortion analysis.

#### 2.1.3 MagPhan RT phantom

The MagPhan Phantom (The Phantom Laboratory) consists of two pieces, top and bottom, each weighing 12 kg. It is filled with a water‐based solution composed of 96.4 % distilled water, 2.5 % polyvinylpyrrolidone (PVP), sodium chloride, potassium sorbate, copper sulfate, and blue food coloring. The phantom contains 500 spherical fiducials, each 10 mm in diameter, used to assess geometric distortions within a 350 mm × 270 mm × 210 mm field of view in the left–right, anterior–posterior, and head–foot orientations.

The phantom can be rotated by 90°, swapping the 350 mm dimension with the 210 mm dimension. In addition to evaluating distortion, it measures signal uniformity, SNR, slice thickness along the three cardinal axes, and resolution in each axis. It also assesses laser alignment.

#### 2.1.4 Sun nuclear large field MRI distortion phantom

The Sun Nuclear Large Field MRI Distortion Phantom (Sun Nuclear Corporation) is designed to measure geometric distortions over a clinically relevant, large‐field imaging volume. Constructed from PMMA (acrylic), it is shipped empty and intended for filling with a water‐based solution to create a more tissue‐like environment. The phantom weighs approximately 7.7 kg when empty and about 28.1 kg when filled. Its outer dimensions are 300 mm × 276 mm × 300 mm.

An internal orthogonal 3D grid features intersections marked by 6 mm diameter spheres, with nominal grid spacing of 20.3 mm (inferior–superior), 20.5 mm (anterior–posterior), and 21.5 mm (left–right). Alignment markings facilitate setup, and the design accommodates flat MRI‐Linac couches. However, this phantom can be used to measure spatial distortion.

### 2.2 General scan setup

**FIGURE 1:**
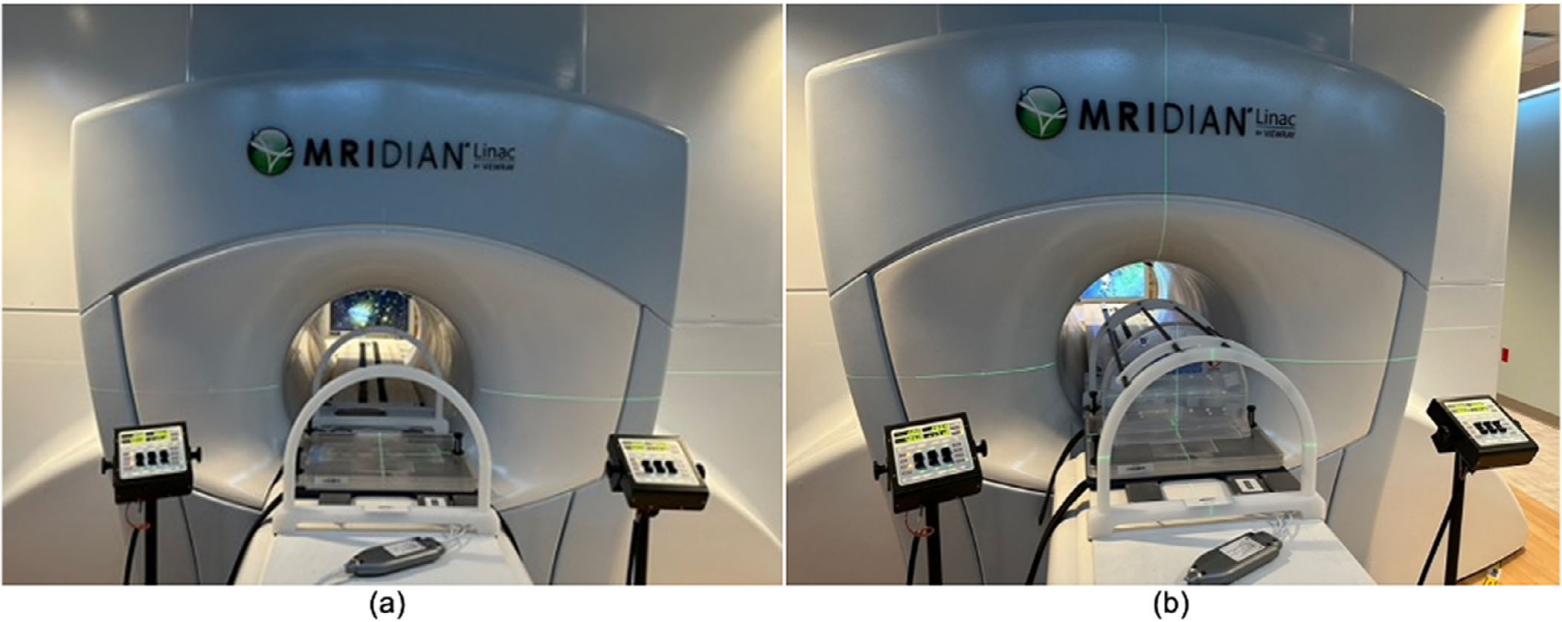
The QUASAR Insight Phantom positioned at the virtual isocenter prior to being moved to the treatment isocenter. The phantom consists of two main parts: (a) horizontal (base) plate and an upright plate, along with a coil bridge accessory. One torso coil is placed beneath the bridge, and the other is placed above it (not shown) before the phantom is transferred. Panel (a) shows the base plate, while panel (b) shows the upright plate mounted on the base, illustrating the axial configuration.

**FIGURE 2:**
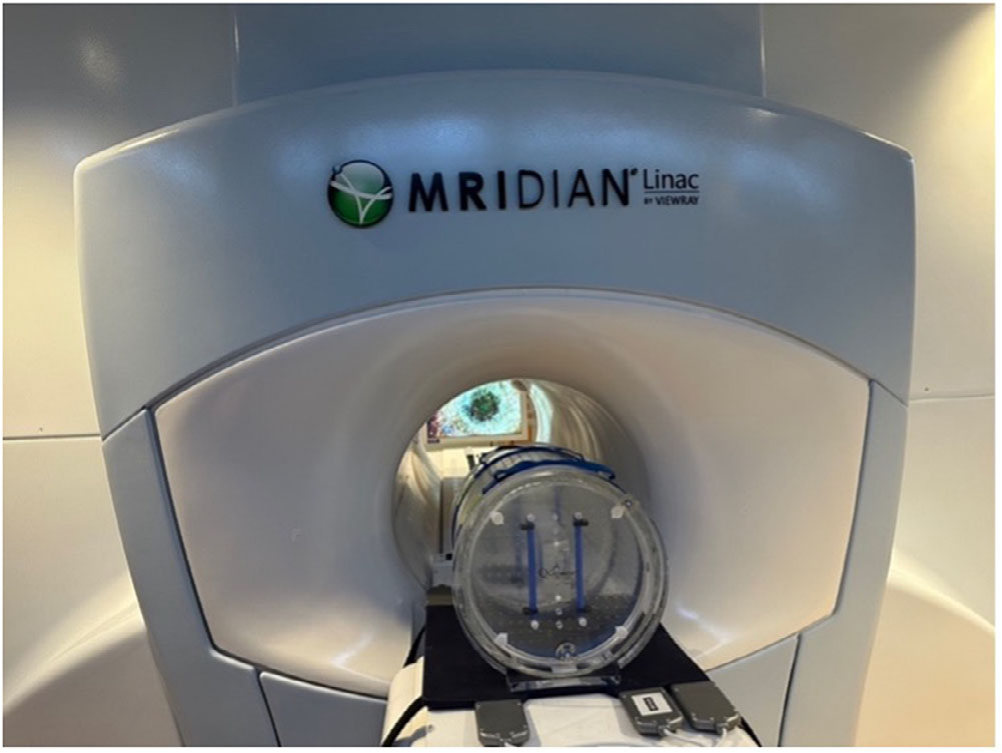
QUASAR MRID^3D^ Geometric Distortion Phantom positioned at the virtual isocenter with the torso coil underneath. Top torso coil not shown in the picture.

**FIGURE 3:**
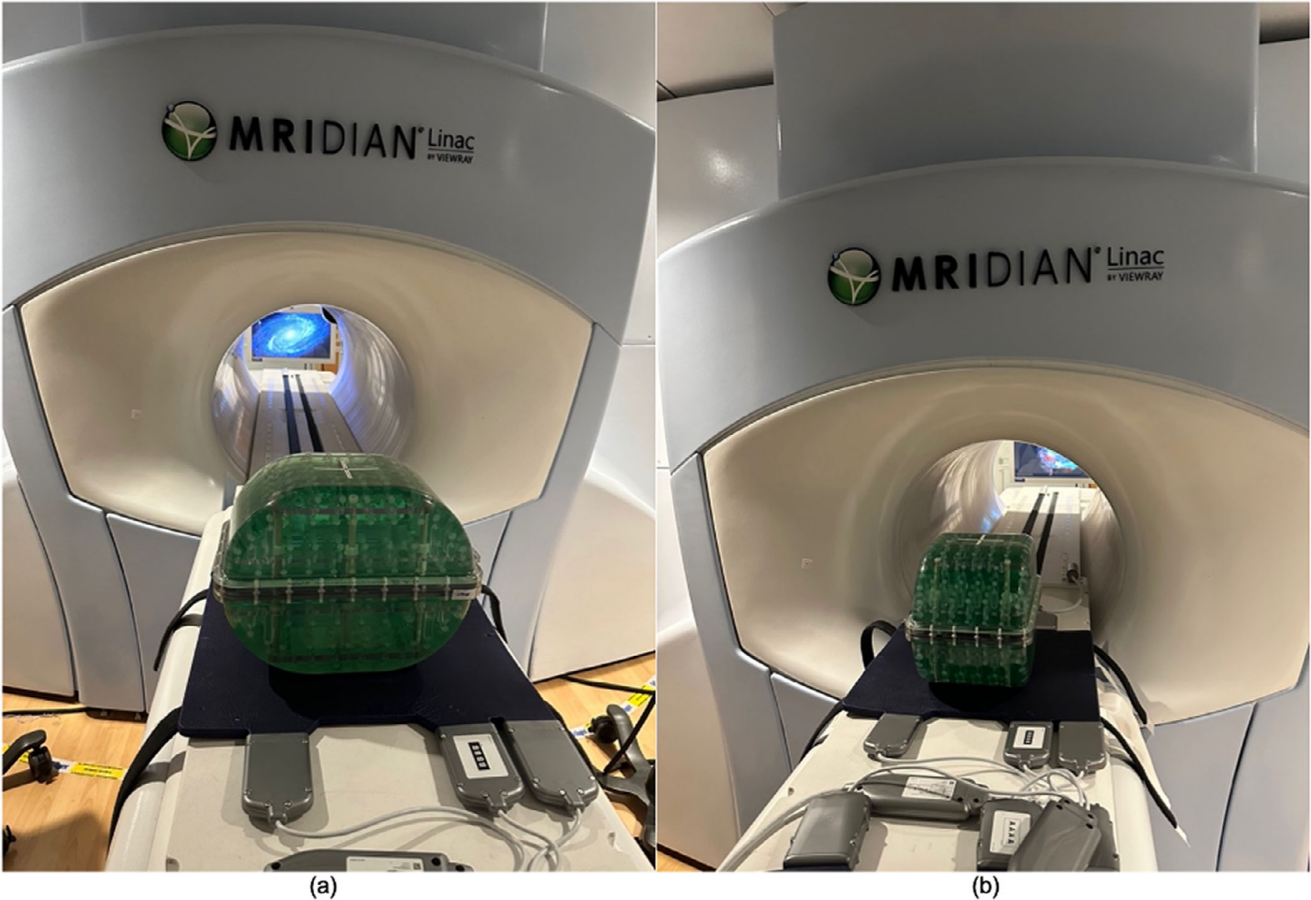
MagPhan Phantom at the virtual isocenter with the lower torso coil beneath it (Top torso coil not shown in the picture). (a) The phantom is oriented laterally, measuring 350 mm (left–right), 270 mm (anterior–posterior), and 210 mm (head–foot), which was used in this study. (b) The phantom is rotated 90°, swapping the 350 mm dimension with the 270 mm dimension. This orientation was not used here but is shown for illustration.

**FIGURE 4:**
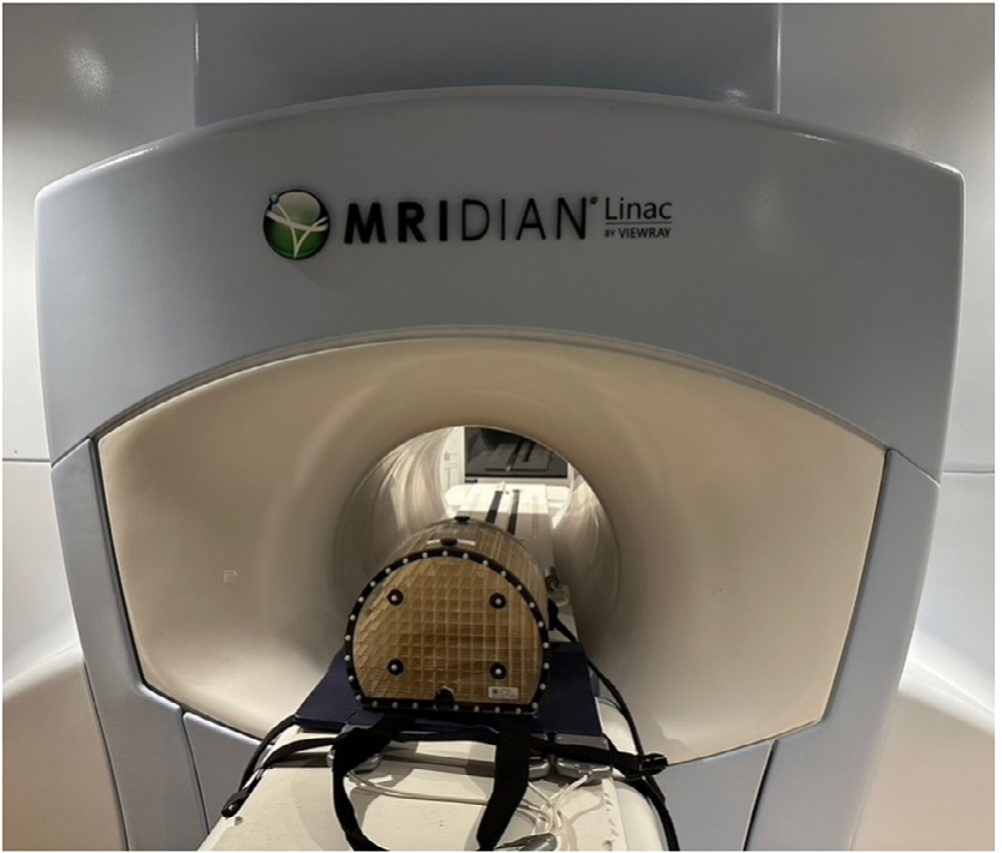
Sun Nuclear Large Field MRI Distortion Phantom at the virtual isocenter with the lower torso coil beneath it. (Top torso coil not shown in the picture).

All phantoms were scanned on a single 0.35 T MR‐Linac unit. Each was positioned on the couch at the virtual isocenter for initial alignment, with the torso coil placed beneath and on top of the phantom. The setup was then moved to the treatment isocenter inside the 0.35 T MR‐Linac bore, located 155 cm from the virtual isocenter. The setups of the phantoms are shown in Figure 1 (QUASAR Insight Phantom), Figure 2 (QUASAR MRID^3D^ Phantom), Figure 3 (MagPhan Phantom), and Figure 4 (Sun Nuclear Large Field MRI Distortion Phantom).

### 2.3 3D TRUFI MR scan procedure

Prior to scanning the four phantoms, the MRI system was shimmed to optimize B_0_ homogeneity. An initial MRI scan then ensured accurate phantom positioning at the MRI isocenter and confirmed that the field of view fully encompassed each phantom. The clinical scanning mode for the 3D MRI acquisition was the TRUFI sequence supplied by the 0.35 T MR‑Linac manufacturer.

The QUASAR Insight Phantom was scanned separately in the axial, sagittal, and coronal orientations to capture the specific image‐quality parameters each orientation provides. The MagPhan Phantom was scanned with a lateral placement to record its corresponding image‐quality metrics. The QUASAR MRID^3D^ Geometric Distortion Phantom required two scans: one with phase encoding from anterior to posterior (A→P) and another with phase encoding reversed—from posterior to anterior (P→A)—by applying a 180° phase shift, as recommended by the manufacturer to enable isolation and evaluation of gradient nonlinearity and B_0_ distortions. To ensure consistency, the phantom's position remained unchanged between the two scans.

Table 1 outlines the TRUFI sequence parameters, listing both the scientific terminology and the Siemens system nomenclature used in the 0.35 T MR‐Linac. All phantoms were imaged at gantry angles from 0° to 360°, in 30° increments. A scan time of 172 s was used for the QUASAR Insight Phantom, the QUASAR MRID^3D^ Phantom, and the MagPhan Phantom, while the Sun Nuclear Large Field MRI Distortion Phantom was scanned in 128 s.

**TABLE 1:** Nomenclature and acquisition parameters for the TRUFI clinical sequence in the 0.35 T MR‐Linac.

		Acquisition time (s)	
Parameter	Nomenclature in 0.35T MR‐Linac	128	172
TR (ms)	TR (ms)	3.35	3.37
TE (ms)	TE (ms)	1.45	1.45
Flip angle	Flip angle	60	60
FOV (mm^3^)	FoV read (mm^3^)	400	449.1
	Adjustment F > > H	350	350
Matrix	Base resolution	266 × 266	300 × 334
	Slab thickness	432	432
	Slice thickness (mm)	1.5	1.5
rBW (Hz/Px)	Bandwidth (Hz/Px)	537	535
NSA	Average	1	1

## 3 RESULTS

### 3.1 IBA QUASAR MRgRT insight phantom

**FIGURE 5:**
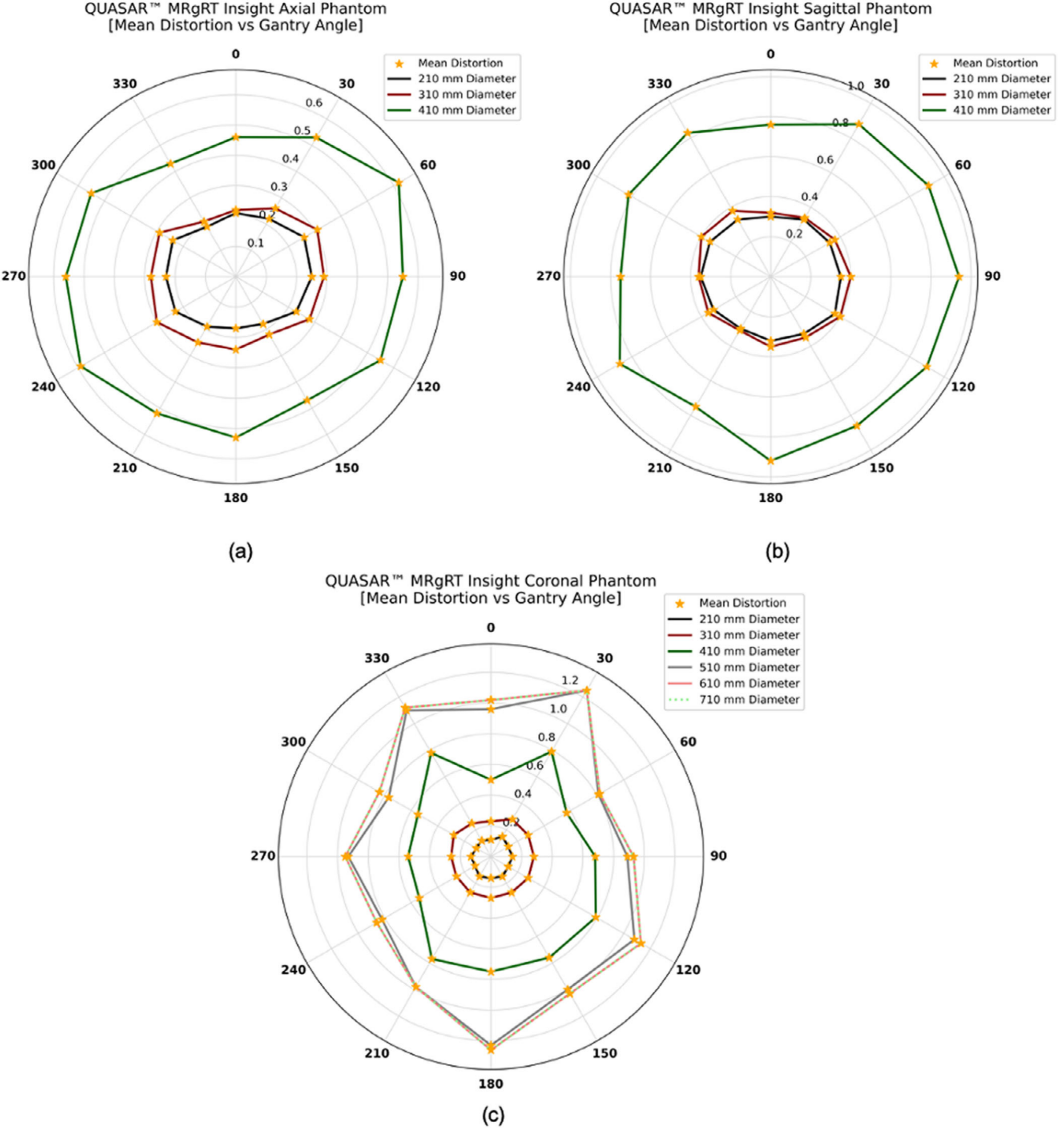
Shows radar plots of the mean distortions for gantry angles from 0° to 360°. Panel (a) illustrates the axial section, panel (b) the sagittal section, and panel (c) the coronal section. Different diameters: 210 mm (black), 310 mm (dark red), 410 mm (dark green), 510 mm (dark gray), 610 mm (light coral), and 710 mm (light green) are color‐coded, with orange star markers denoting individual distortion measurements.

Figure 5 displays the mean distortion (combined gradient nonlinearity and B_0_ effects) measured from 0° to 360° in the axial, sagittal, and coronal sections of the QUASAR Insight Phantom. In general, mean distortion increases with diameter: larger diameters exhibit more pronounced variation at certain gantry angles, while smaller diameters cluster more closely. In Figure 5a (axial section), the radar plot shows mean distortion values of 0.22 ± 0.03 mm at a 210 mm diameter, 0.26 ± 0.04 mm at 310 mm, and 0.53 ± 0.05 mm at 410 mm, averaged over all gantry angles. In Figure 5b (sagittal section), mean distortions are somewhat higher at 0.33 ± 0.02 mm, 0.36 ± 0.03 mm, and 0.85 ± 0.07 mm for 210, 310, and 410 mm diameters, respectively, averaged over all gantry angles. In Figure 5c (coronal section), six diameters (210, 310, 410, 510, 610, and 710 mm) are evaluated, yielding mean distortions of 0.13 ± 0.01, 0.27 ± 0.02, 0.67 ± 0.12, 0.98 ± 0.16, 1.02 ± 0.15, and 1.02 ± 0.15 mm, respectively, averaged over all gantry angles.

Table 2 summarizes the results from the QUASAR Insight Phantom, revealing notable differences among the axial, sagittal, and coronal sections. The coronal section had the highest uniformity (89.60), whereas the axial section showed the highest SNR (300.94). Image SNR showed higher values in the axial and coronal orientations. Modulation and MTF were greatest in the axial orientation. The measured slice thickness exceeded the nominal 1.5 mm in all orientations but approached 1.97 mm in the coronal view. Positional offsets and twist angles were minimal across all axes, indicating only slight deviations.

**TABLE 2:** Parameters measured for the QUASAR Insight Phantom in the axial, sagittal, and coronal sections. All measurements were performed at a gantry angle of 300°.

		Scan section		
Parameter		Axial	Sagittal	Coronal
**Uniformity**		65.17	60.23	89.60
**SNR**		300.94	50.25	253.14
**Image SNR**		463.72	91.82	469.89
**Modulation**		0.47	0.41	0.25
**MTF**		0.71	0.62	0.37
**Measured thickness (mm)**		2.32	2.37	1.97
**Position (mm)**	X	0.25	−0.20	0.03
	Y	0.36	0.24	0.29
	Z	−0.91	−1.12	−0.87
**Twist (deg)**	X	1.15	0.38	−0.33
	Y	0.79	−0.31	0.24
	Z	−0.06	0.61	0.60

### 3.2 QUASAR MRID^3D^ geometric distortion phantom

**FIGURE 6:**
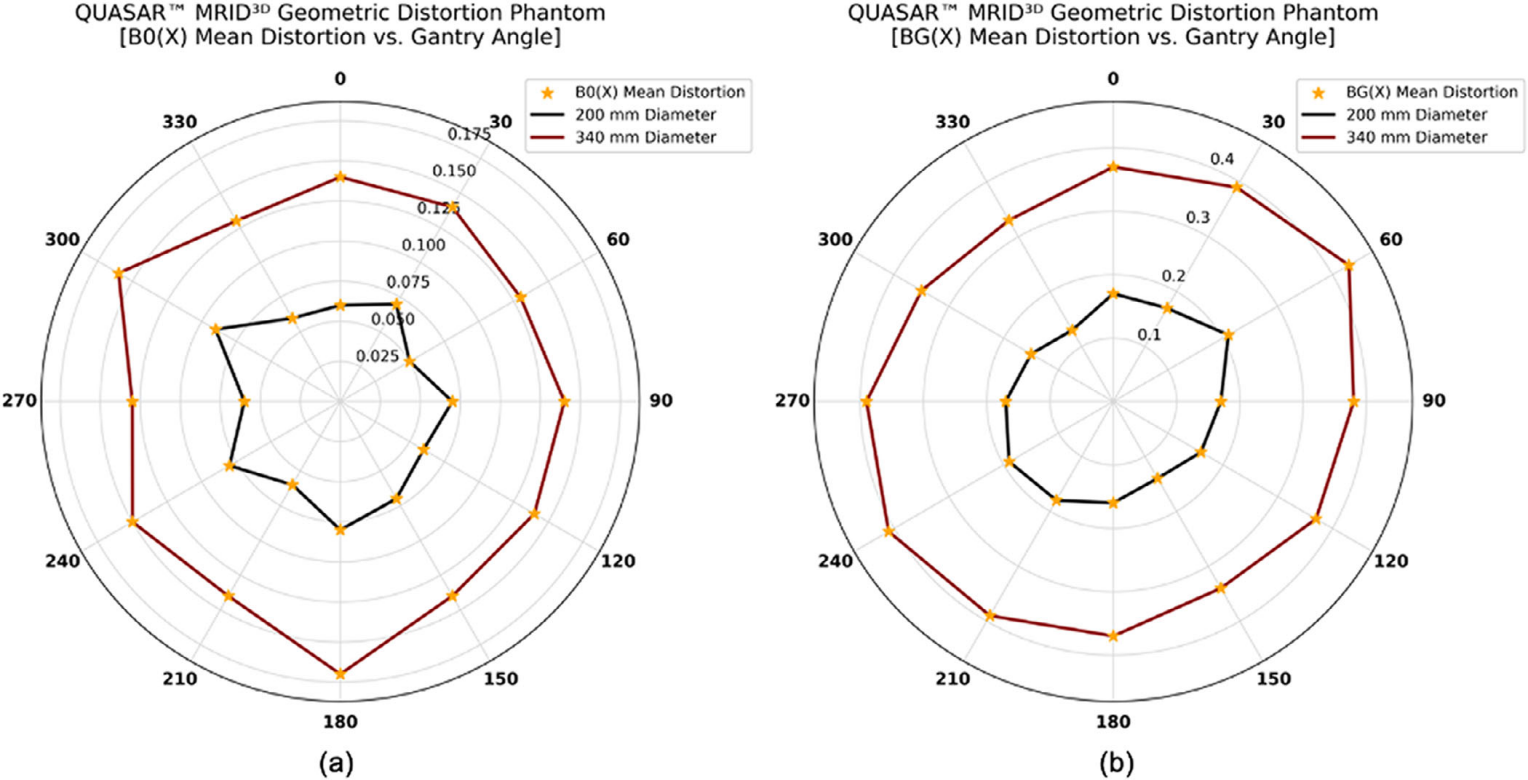
Shows mean distortion (0°–360°) measured with the QUASAR MRID^3D^ phantom for (a) B_0_(x) and (b) BG(x). Distortion is reported at diameters of 200 mm (black) and 340 mm (dark red). Orange stars indicate individual measurements at each angle, and connecting lines are shown only for visualization.

Figure 6 presents mean distortion measurements from the QUASAR MRID^3D^ phantom at various gantry angles, comparing the primary magnetic field B_0_(x) to the gradient magnetic field BG(x). Two diameters (200 and 340 mm) were assessed. As shown in Figure 6a, the B_0_(x) mean distortion at a 200 mm diameter ranged from 0.05 to 0.09 mm, averaging 0.07 ± 0.01 mm. At 340 mm, distortion values ranged from 0.13 to 0.17 mm, with an average of 0.14 ± 0.01 mm. For BG(x) (Figure 6b), distortions at a 200 mm diameter varied from 0.13 to 0.21 mm, yielding 0.17 ± 0.02 mm on average, while at 340 mm they ranged from 0.33 to 0.43 mm, with an average of 0.38 ± 0.03 mm.

### 3.3 MagPhan RT phantom

**FIGURE 7:**
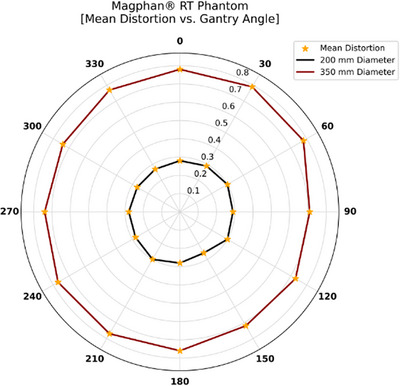
Illustrates mean distortion (in mm) versus gantry angle for the MagPhan Phantom, recorded at 200 mm diameter (black) and 350 mm diameter (red). Orange stars represent the measured values, and connecting lines are shown only for visualization.

Figure 7 shows the measured mean distortion (combined gradient nonlinearity and B_0_ effects) for the MagPhan Phantom at gantry angles ranging from 0° to 360°. At a 200 mm diameter, distortion was relatively low (0.26 to 0.30 mm), averaging 0.28 ± 0.01 mm. In contrast, at 350 mm it ranged from 0.71 to 0.79 mm, with an overall average of 0.76 ± 0.03 mm, indicating that distortion increases with distance from the MRI isocenter. Additional MRI parameters are listed in Table 3, including an SNR of approximately 50 and a measured slice thickness of 2.4 mm. Uniformity values reached 308 at a 150 mm diameter and 447 at 300 mm. MTF analysis showed an edge spread function (ESF) 10%–90% transition width of 1.91 mm in columns and 1.57 mm in rows, while the FWHM measured 2.27 mm horizontally and 2.00 mm vertically, reflecting strong image sharpness. For laser alignment, minor translational offsets were noted at −1.2 mm (left), −1.7 mm (anterior), and −0.9 mm (head). Angular deviations for yaw, pitch, and roll were −0.27°, −0.07°, and 0.07°, respectively.

**TABLE 3:** Measured parameters and sub‐parameters for the MagPhan Phantom. All measurements were performed at a gantry angle of 300°.

Parameter	Sub‐parameter	Value
**SNR**	(none)	50
**Slice thickness (mm)**	(none)	2.4
**Uniformity**	150 mm diameter	308
	300 mm diameter	447
**MTF analysis**	**Edge spread function (ESF) 10%–90% Transition width**:	
	Column width (mm)	1.91
	Row width (mm)	1.57
	**PSF Full‐Width‐Half‐Max (FWHM)**:	
	Horizontal FWHM (mm)	2.27
	Vertical FWHM (mm)	2
**Laser Alignment**	**Translational offsets**:	
	Left (mm)	−1.2
	Anterior (mm)	−1.7
	Head (mm)	−0.9
	**Angular deviation**:	
	Yaw	−0.27
	Pitch	−0.07
	Roll	0.07

### 3.4 Sun nuclear large field MRI distortion phantom

**FIGURE 8:**
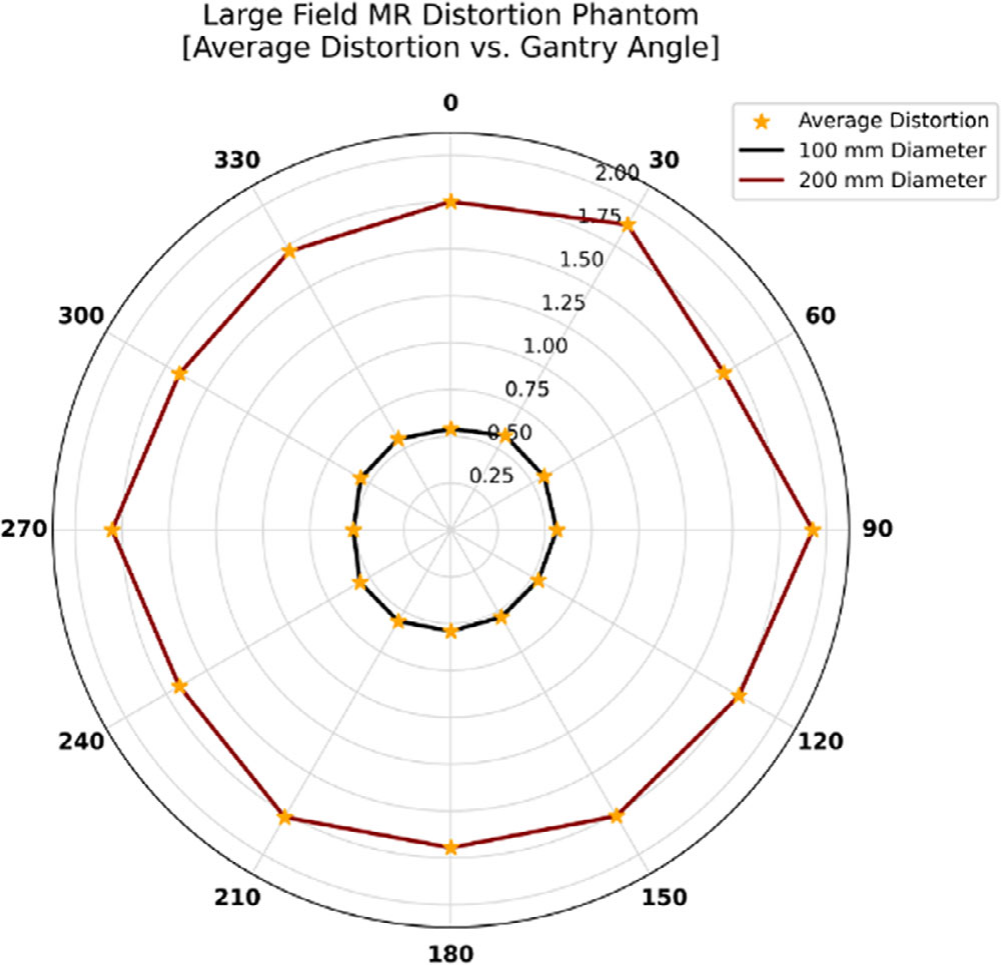
Average distortion measurements for the Sun Nuclear Large Field MRI Distortion Phantom at 100and 200 mm diameters, recorded at gantry angles from 0° to 360° in 30° increments. Orange stars mark the average distortion at each angle.

Figure 8 presents the Sun Nuclear Large Field MRI Distortion Phantom, illustrating average distortions (combined gradient nonlinearity and B_0_ effects) at gantry angles from 0° to 360° in 30° increments, measured at two diameters (100 and 200 mm). For the 100 mm diameter, the minimum average distortion is 0.54 mm and the maximum is 0.581 mm, yielding a mean value of 0.56 ± 0.02 mm. For the 200 mm diameter, the minimum and maximum average distortions are 1.718 and 1.804 mm, respectively, with a mean distortion of 1.76 ± 0.04 mm.

## 4 DISCUSSION

This study conducted a comparative evaluation of four commercially available phantoms: IBRA QUASAR MRgRT Insight Phantom, IBA QUASAR MRID^3D^ Geometric Distortion Phantom, MagPhan Phantom, and the Sun Nuclear Large Field MRI Distortion Phantom, used to measure MRI distortions and other image quality parameters in a 0.35 T MR‐Linac. The Sun Nuclear Large Field MRI Distortion Phantom is reported here for the first time, enriching the comparative analysis.

The clinical MRI protocol used in this study is TRUFI, a type of balanced steady‐state free‐precession (bSSFP) sequence.^24^ TRUFI is particularly advantageous because of its high SNR and excellent temporal resolution. Nevertheless, it is sensitive to B_0_ fluctuations; large variations in B_0_ can produce image artifacts and isocenter shifts.^10^, ^11^ This sensitivity is especially relevant in the 0.35 T MR‐Linac design, where the Linac is positioned between two MRI magnets, making such distortions and isocenter shifts more pronounced as the gantry rotates.

To characterize distortions, we evaluated gantry angles in 30° increments from 0° to 360° for all four phantoms. Each phantom's software provided mean distortion values at specific diameters. The MagPhan Phantom reported mean distortion at 200 and 350 mm diameters, while the QUASAR MRID^3D^ Phantom separately quantified B_0_‐induced distortions and gradient nonlinearity distortions at 200 and 340 mm diameters. In the QUASAR Insight Phantom, diameters varied by phantom section: 210, 310, and 410 mm in the axial and sagittal sections, and 210 mm up to 710 mm in the coronal section. For the Sun Nuclear Large Field MRI Distortion Phantom, although the software assesses a range of 5–215 mm, we plotted results at 100 and 200 mm to maintain consistency across phantoms.

All four phantoms demonstrated that mean distortions in the 0.35 T MR‐Linac depend on gantry angle. However, all of the phantoms exhibited distortion values that fell within the 0.35 T MR‐Linac's acceptance specifications: <1 mm across a 20 cm FOV and <2 mm across a 35 cm FOV. The QUASAR MRID^3D^ Phantom measures two distortion components: the average B_0_‐induced distortion (0.07 ± 0.01 mm at 200 mm and 0.14 ± 0.01 mm at 340 mm) and gradient nonlinearity distortion (0.17 ± 0.02 mm at 200 mm and 0.38 ± 0.03 mm at 340 mm). The MagPhan Phantom showed higher overall values (0.28 ± 0.01 mm at 200 mm and 0.76 ± 0.03 mm at 350 mm). The QUASAR Insight Phantom, with its multiple sections, yielded distortion values that varied by orientation. In the coronal section, the average mean distortion across all gantry angles was 0.13 ± 0.01, 0.27 ± 0.02, 0.67 ± 0.12, 0.98 ± 0.16, 1.02 ± 0.15, and 1.02 ± 0.15 mm at diameters of 210, 310, 410, 510, 610, and 710 mm, respectively. In the axial section, these values were 0.22 ± 0.03 mm at 210 mm, 0.26 ± 0.04 mm at 310 mm, and 0.53 ± 0.05 mm at 410 mm. The sagittal section displayed even higher distortion: 0.33 ± 0.02 mm, 0.36 ± 0.03 mm, and 0.85 ± 0.07 mm at 210, 310, and 410 mm, respectively, underscoring how distortions differ across coronal, axial, and sagittal planes. This pattern likely reflects the 0.35 T MRI‐Linac's design, in which two “doughnut” magnets accommodate the Linac. In the Sun Nuclear Phantom, the mean distortion was 0.56 ± 0.02 mm at a 100 mm diameter and 1.76 ± 0.04 mm at 200 mm, representing the highest values measured. This could be attributed to the phantom's grid design and use of distilled water rather than a high‐contrast fluid. However, in general, distortion depends on the phantom design, the material used to fill the phantom, and the diameter and orientation used to scale the distortion; as the diameter increases, the distortion also increases.

Among the four phantoms, MagPhan and QUASAR Insight also quantify various image quality parameters. For instance, the MagPhan Phantom measured an SNR of 50, whereas the QUASAR Insight Phantom reported SNR values of 50.25 in the sagittal plane, 253.14 in the coronal plane, and 300.94 in the axial plane (See Table 2 and Table 3). These differences can be partially explained by each phantom's design and its positioning relative to the torso coil array. In uniformity measurements, MagPhan values of 308 and 447 at 150 and 300 mm diameters, respectively, differ from the QUASAR Insight Phantom's 65.70 (axial), 60.23 (sagittal), and 89.60 (coronal). These are not strictly comparable across phantoms because each uses a distinct scale. The MagPhan Phantom also provides detailed MTF analysis, reporting ESF and PSF values in horizontal and vertical planes, whereas the QUASAR Insight Phantom presents single MTF values in each orientation.

For laser alignment and phantom positioning, the MagPhan and QUASAR Insight phantoms report lateral, vertical, and longitudinal offsets, as well as yaw, pitch, and roll angles (Table 2 and Table 3). The MagPhan, lacking flat surfaces, can tilt during setup, causing higher reported offsets than the QUASAR Insight Phantom, which has flat surfaces to ensure more stable placement. The QUASAR MRID^3D^ Phantom showed a vertical offset (11.94 mm) due to the phantom's internal reference cross not aligning precisely with the lasers.

**FIGURE 9:**
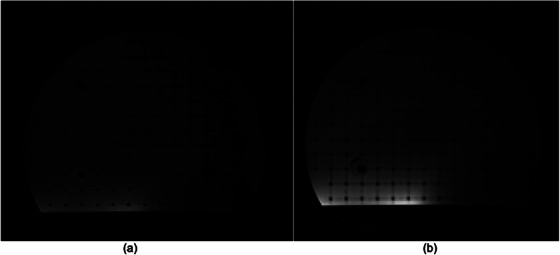
Presents MRI images of the Sun Nuclear Large Field MRI Distortion Phantom under two TRUFI protocols, illustrating the effect of scan time and FOV on image quality. In (a), the 172‑s scan produces visible artifacts at the phantom's edges, hindering distortion analysis. In (b), these artifacts are minimized with a 128‑s scan, making distortion analysis possible. The different scan times reflect varying FOV configurations.

The TRUFI sequence offers multiple protocols with varying FOV and scan times (65, 92, 128, and 172 s). In this study, we compared four phantoms using the TRUFI sequence with parameters detailed in Table 1. Three of the phantoms—the QUASAR Insight Phantom, QUASAR MRID^3D^ Geometric Distortion Phantom, and MagPhan Phantom—were imaged using a 172 s protocol to ensure a sufficiently large field of view encompassing each phantom's entire volume. However, this largest FOV protocol was not suitable for the Sun Nuclear Large Field MRI Distortion Phantom, as the broader coverage introduced edge artifacts that interfered with the analysis (see Figure 9). Given that the voxel size and slice thickness remained constant across protocols, the primary difference among the series was the number of slices acquired above and below the inferior and superior extents of the phantom. By selecting a 128 s protocol, we reduced the excess slice coverage, thereby mitigating artifacts and better aligning the frame of reference (i.e., the original CT model of the phantom) with the MRI imaging volume. This approach effectively eliminated problematic edge artifacts for the Sun Nuclear Phantom, as illustrated in Figure 9. However, further improvements and additional assessments of the Sun Nuclear Large Field MRI Distortion Phantom are recommended.

## 5 CONCLUSION

Four commercially available phantoms: the QUASAR Insight Phantom, the QUASAR MRID^3D^ Phantom, the MagPhan Phantom, and the Sun Nuclear Large Field MRI Distortion Phantom, were evaluated on a single 0.35 T MR‐Linac to compare distortion, assess image quality parameters, and examine the impact of gantry rotation and phantom orientation. All phantoms demonstrated that B_0_ and gradient‐induced distortions vary with gantry rotation, with the extent of distortion influenced by phantom design, fiducial geometry, and analysis software. Image quality metrics differed among the phantoms based on their respective designs and analysis methods.

## AUTHOR CONTRIBUTIONS

**Mateb Al Khalifa**: Conceptualization; acquisition; interpretation of data; methodology; validation; drafting the work; revising the draft; final approval of the version. **Tianjun Ma**: Revising the draft; final approval of the version. **Haya Aljuaid**: Revising the draft; final approval of the version. **Siyong Kim**: Revising the draft, final approval of the version. **William Y. Song**: Revising the draft; final approval of the version.

## CONFLICT OF INTEREST STATEMENT

The authors declare no conflicts of interest.
